# Comparative Evaluation of a Disinfectant Formulation Comprising Hydrogen Peroxide, Peracetic Acid, and Acetic Acid Against *Aspergillus niger*, *Escherichia coli*, and *Staphylococcus aureus* on Various Surfaces in Comparison to Other Disinfectants

**DOI:** 10.1155/ijm/2602317

**Published:** 2025-06-24

**Authors:** Md. Raisul Islam Rabby, Md. Sabbir Hossain, Nafisa Nusrat Chowdhury, Fatema Akter, Mamudul Hasan Razu, Zabed Bin Ahmed, Mala Khan

**Affiliations:** Bangladesh Reference Institute for Chemical Measurements (BRiCM), Dhaka, Bangladesh

## Abstract

This study investigates the effectiveness of a disinfectant formulation comprising acetic acid, hydrogen peroxide, and peracetic acid (AAHPA) against *Aspergillus niger*, *Escherichia coli*, and *Staphylococcus aureus* on petri plates, smooth surfaces, and rough surfaces. Comparative analysis with conventional disinfectants such as 70% isopropyl alcohol, 1.5% chlorhexidine gluconate, 10% sodium hypochlorite, and 0.2% hydrogen peroxide reveal AAHPA's superior performance, achieving significantly higher log reduction (*p* < 0.05) and percentage reduction (*p* < 0.01) against *A. niger* in 5 min on petri plates. On smooth and rough surfaces, AAHPA exhibits exceptional efficacy, demonstrating the highest log and percentage reduction against *A. niger* in 0.5, 1, and 5 min, surpassing other disinfectants. AAHPA shows the highest bacterial decline against *E. coli* and *S. aureus*, followed by 0.2% hydrogen peroxide. In some cases, 0.2% hydrogen peroxide was slightly more effective than AAHPA against *E. coli* and *S. aureus* in 5 min. Time-dependent analysis of log reduction trends emphasizes AAHPA's rapid and consistent effectiveness, particularly in 0.5 min, positioning it as a promising disinfectant formulation with broad-spectrum efficacy across diverse surfaces.

## 1. Introduction

Disinfection is the process of eliminating almost all harmful organisms from inanimate objects and surfaces, reducing microbial contamination to a safe level [1]. Disinfectants are widely used in food processing, farming, healthcare facilities, residential, and pharmaceutical products [2]. Due to decades of disinfectant use, there is now a diverse range of agents available with varying levels of antimicrobial activity [3]. Several chemical agents, such as iodine compounds, chlorine compounds, hydrogen peroxide (H_2_O_2_), peroxy acids, glutaraldehyde, ethylene oxide, formaldehyde, and beta propiolactone have biocidal and sporicidal activity [4].

Chlorine has been extensively employed as a disinfectant targeting bacterial spores [4]. However, the use of chlorine poses challenges due to issues like the accumulation of derivatives and instability in the presence of organic matter [5]. This situation prompts exploration into innovative and ecofriendly compounds that can serve as alternatives to chlorine. Peracetic acid (PAA) is widely utilized and stands as a viable substitute for chlorine-based compounds in disinfection processes [6]. PAA disinfectants, known for their environmentally friendly nature, function as broad-spectrum antimicrobials with sporicidal properties. PAA- and H_2_O_2_-based sanitizers are examples of oxidizing agents that can be used individually or in combination to maximize synergistic effects [7–9]. The equilibrium reaction of H_2_O_2_ and acetic acid (AA) results in the formation of PAA and water (H_2_O_2_+CH_3_COOH ⇌ CH_3_COOOH+H_2_O). As it is a bidirectional reaction, PAA and water undergo a reaction to regenerate H_2_O_2_ and AA. When the system reaches an equilibrium state, the rates of these reactions become equal, resulting in no further change in the concentrations of the PAA, AA, and H_2_O_2_ [10]. Constant concentrations of these chemicals can contribute to the effective disinfection. AA plays a crucial role in stabilizing the PAA solution. This combination not only stabilizes the disinfectant solution but also enhances its sporicidal properties [8]. While H_2_O_2_ contributes to the disinfection capacity of the PAA mixture, PAA is considered a more potent antimicrobial agent than H_2_O_2_. As a single chemical, both PAA and H_2_O_2_ need far higher doses than the combination of these two chemicals to achieve the same amount of disinfection [8, 11]. Several products comprising the combination of PAA and H_2_O_2_ have received approval from the U.S. Food and Drug Administration for use as high-level disinfectants [12]. They are utilized in diverse sectors, including the food industry, water treatment, medical instrument disinfection, and synthetic chemistry [13–16]. The antimicrobial activity of PAA is dependent on time and dose, making it suitable for applications in wastewater treatments and healthcare disinfection [17–19]. In healthcare, PAA is considered a chlorine substitute, especially effective against clinical strains resistant to cephalothin, chloramphenicol, and trimethoprim, which exhibit chlorine tolerance [20]. The byproducts of PAA disinfection are less toxic compared to those of chlorine-based disinfectants, minimizing their impact on the ecosystem [21].

The use of disinfectants and other antimicrobial agents exerts a selective pressure on both commensal and pathogenic bacteria which leads to resistance to these agents. Resistance can arise from intrinsic or acquired resistance mechanisms through horizontal and vertical gene transfer [22, 23]. A study on 510 *Escherichia coli* isolates investigated their susceptibility to four disinfectants—cetyltrimethylammonium bromide, benzalkonium chloride, cetylpyridinium chloride, and chlorhexidine on 10 disinfectant-resistant genes. All disinfectants showed high minimal inhibitory concentrations up to 2048 mg/L [24]. A recent antifungal susceptibility investigation showed that planktonic cells of *Candida albicans* were completely resistant to benzalkonium chloride [25]. This raises concerns about the rapid emergence of disinfectant resistance, as fungi and bacteria are showing less sensitivity to conventional disinfectant agents [23, 25–27]). In both outbreak and endemic settings, surface disinfection is recommended as part of comprehensive measures to control nosocomial transmission [28–30]. Processes aimed at sterilizing food, pharmaceuticals, and medical products have to consider this high level of resistance as a critical factor.

A detailed observation on decontamination, disinfection, and sterilization suggested more research and new methods of cleaning and disinfection are needed [31]. As the world is searching for effective disinfectants against disinfectant-resistant microorganisms and to substitute chlorine-based agents, this study is aimed at observing the comparative effectiveness of a disinfectant formulation comprising acetic acid, hydrogen peroxide, and peracetic acid (AAHPA) with some conventional disinfectant agents. The study will contribute to the development of effective disinfection strategies across diverse applications, promoting a safer and cleaner environment in healthcare, industry, and other relevant settings.

## 2. Materials and Methods

### 2.1. Microorganisms

One fungal strain and two bacterial strains were taken for the study from the American Type Culture Collection (ATCC) (Microbiologics, Minnesota, United States). The fungal strain was *Aspergillus niger* ATCC 16888⁣^∗^, and the bacterial strains were *E. coli* ATCC 25922⁣^∗^ and *Staphylococcus aureus* subsp. *aureus* ATCC 6538⁣^∗^.

### 2.2. Spore Suspension Preparation of *A. niger*

To collect spores, *A. niger* ATCC 16888⁣^∗^ stock culture was flooded with autoclaved distilled water, and spores were gently released by brushing with an inoculation needle. The spore suspension was centrifuged for 5 min at 500 × g to sediment any hyphae or conidiophores, followed by wash-through centrifugation for 10 min at 12, 000 × g. The collected spores were resuspended in 200 *μ*L autoclaved distilled water in microcentrifuge tubes, followed by thorough vortexing to break up any clumps of spores [32].

### 2.3. Preparation of Bacterial Suspension

Transferred one loopful of colonies of *E. coli* ATCC 25922⁣^∗^ and *S. aureus* subsp. *aureus* ATCC 6538⁣^∗^ culture stock solution (approximately 100 *μ*L) on Tryptic Soy Agar (TSA) medium and incubated the plate at 37°C for at least 24 h (a first incubation). After the first incubation was completed, a loopful of the colonies was collected by using a platinum inoculation loop from a TSA plate and transferred into 2 mL 0.1% peptone broth. After the incubation of the peptone broth at 37°C for at least 24 h (a second incubation), 100 *μ*L bacterial strain suspensions were spread on the TSA using the spread plating method.

### 2.4. Disinfectant Formulation

A broad-spectrum disinfectant agent was formulated by mixing 2.65% AA and 3% H_2_O_2_. This mixture undergoes a bidirectional reaction, forming 0.1% PAA. At equilibrium, the final formulation contains AAHPA in a stable state. The range of pH was between 2.5 and 2.9, and the liquid was clear and colorless.

### 2.5. Collection of Conventional Disinfectants

Several disinfectants were purchased from the local market. The collected disinfecting agents were 70% isopropyl alcohol (IPA), 1.5% chlorhexidine gluconate (CHG), 10% sodium hypochlorite (NaOCl), and 0.2% H_2_O_2_.

### 2.6. Disinfectant Efficacy Test

#### 2.6.1. Efficacy Test on Petri Plates

##### 2.6.1.1. Preparation of Adhered Petri Plates

One hundred microliters of bacterial strain suspensions and fungal spore suspension was placed on the TSA and Sabouraud dextrose agar (SDA) plate, respectively, using the spread plating method and air dried.

##### 2.6.1.2. Application Procedure of Disinfectants

The formulated disinfectant agent containing AAHPA and other locally collected disinfectants was applied onto the culture-adhered petri plate. The three distinct evaluated contact times were 0.5, 1, and 5 min, and after that, 2 mL of neutralizer (0.03% sodium thiosulfate solution) was applied to the petri dishes. Thereafter, the adherent bacterial strain and spore were scraped off from the surface of the petri dish using the tip of the pipette. To create the first stage diluted solution, 0.1 mL of the recovered solution (scraped and collected adherent bacteria) was poured into 0.9 mL of saline solution (0.85%) and thoroughly mixed. Using the same solution, this process was repeated to prepare a series of 10-fold dilutions up to six times (~10^6^ dilutions), and in this way, six different dilution samples (10^−1^, 10^−2^, 10^−3^, 10^−4^, 10^−5^, and 10^−6^ dilutions) were prepared. From each dilution, 0.1 mL of samples was transferred to TSA (for bacteria) and SDA (for fungus) and incubated at 37°C for at least 24 h and 25°C for 5 days, respectively. After the incubation, the number of colonies on each medium was counted.

#### 2.6.2. In-House Standardized Procedure

This procedure was used to conduct a modified quantitative surface disinfection test to evaluate the effectiveness of surface cleaning. The two surface types, rough surfaces (which represented floors, walls, etc.) and smooth surfaces (which represented stainless steel, instrument tables, trolleys, scissors, surgical instruments, etc.) were chosen. The concentration of all disinfectants was adjusted. For each disinfection, six 10 × 10 cm shining stainless steel plates and six ceramic plaster tiles were autoclaved. All surfaces were taken for inoculation with selected microorganisms. From each smooth and rough surface, three surfaces were treated with disinfectant for 0.5, 1, and 5 min duration. The other three surfaces kept untreated which were acted as positive control against each disinfectant-treated surface. Sterile cotton gauzes were swabbed on the three surfaces after 0.5, 1, and 5 min contact time of the disinfectants, respectively, and then the cotton gauzes were dipped into 10 mL of 0.85% NaCl solution separately, and the stock solution was prepared. The stock solution was serially diluted to prepare six different dilutions (10^−1^, 10^−2^, 10^−3^, 10^−4^, 10^−5^, and 10^−6^ dilutions). From each dilution, 0.1 mL of samples were transferred to TSA (for bacteria) and SDA (for fungus), and the plates were incubated at 37°C for 24 h and 25°C for 5 days, respectively. After the incubation, the number of colonies on each medium was counted.

### 2.7. Statistical Analysis

All the results are expressed as a mean of two replicates. All statistical analyses were conducted using Microsoft Excel 2019. One-way analysis of variance (ANOVA) and post hoc test (Tukey's HSD) were performed to determine the significance of disinfectants efficacy at *p* < 0.05 level.

## 3. Results

### 3.1. Microbial Decline

Microbial colonies on agar plates were counted in CFU/mL units both before and after the disinfectants were applied. AAHPA killed the *A. niger* spores, *E. coli*, and *S. aureus* more effectively on all surfaces compared to the other four disinfectants. The findings for the colony forming unit, log reduction, and killing percentage on petri plates, smooth surfaces, and rough surfaces are tabulated in Tables 1, 2, and 3, respectively.

### 3.2. Log and Percentage Reduction of Microbes on Petri Plates

**Figure 1:**
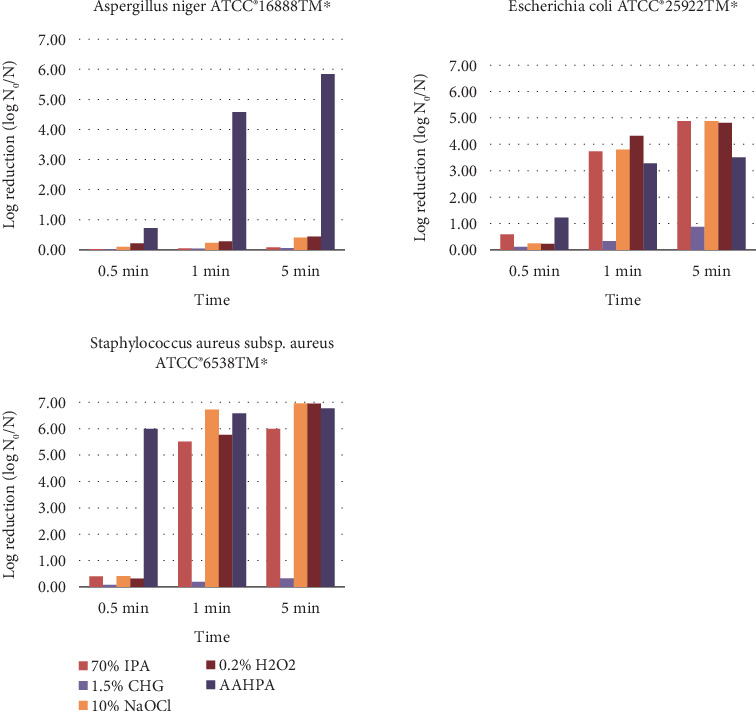
The log reduction of tested microbes and spores by five disinfectants on petri plates at different time intervals.

The log and percentage reduction of tested microbes and spores by five disinfectants on petri plates are tabulated in Table 1. AAHPA showed significantly high log reduction (*p* < 0.05) and percentage reduction (*p* < 0.01) on petri plates against *A. niger* ATCC 16888⁣^∗^. The log reduction of *A. niger* by AAHPA at 5 min was 5.84, where the log reduction by 70% IPA, 1.5% CHG, 10% NaOCl, and 0.2% H_2_O_2_ was 0.079, 0.057, 0.41, and 0.44, respectively (Figure 1). There were no significant differences between the five disinfectants against *E. coli* ATCC 25922⁣^∗^ on the petri plate, where the percentage reduction of *S. aureus* subsp. *aureus* ATCC 6538⁣^∗^ by AAHPA was found to be significant (*p* < 0.05) compared to 1.5% CHG.

### 3.3. Log and Percentage Reduction of Microbes on Smooth Surfaces

**Figure 2:**
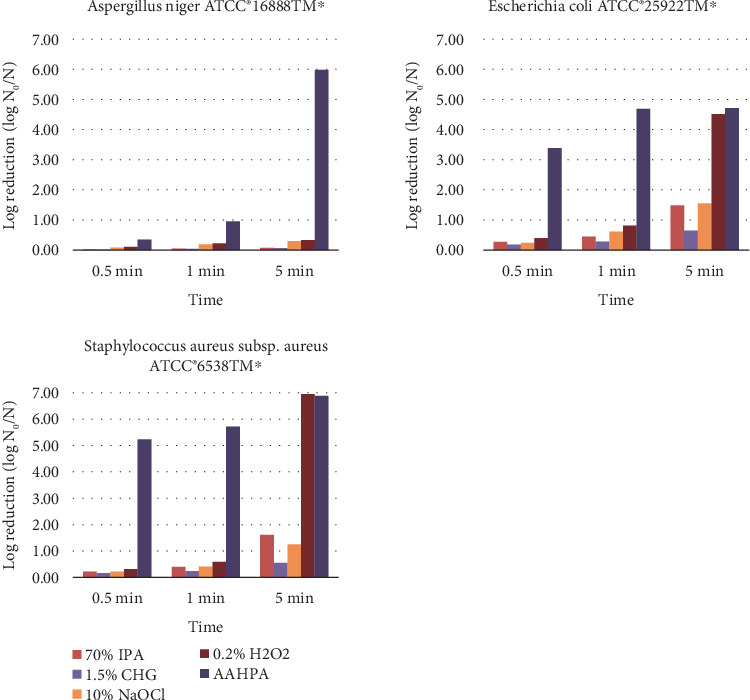
The log reduction of tested microbes and spores by five disinfectants on smooth surfaces at different time intervals.

The log and percentage reduction of tested microbes and spores by five disinfectants on smooth surfaces are tabulated in Table 2. AAHPA showed a significantly high percentage reduction at *p* < 0.01 level compared to 70% IPA and 1.5% CHG and at *p* < 0.05 compared to 10% NaOCl and 0.2% H_2_O_2_ on smooth surfaces against *A. niger* ATCC 16888⁣^∗^. AAHPA showed the highest log and percentage reduction at 0.5 (0.35% and 55.17%), 1 (0.96% and 88.93%), and 5 min (5.99% and > 99.999%) on smooth surfaces compared to all other tested disinfectants against *A. niger* (Figure 2).

0.2% H_2_O_2_ and AAHPA were highly effective against *E. coli* ATCC 25922⁣^∗^ and *S. aureus* subsp. *aureus* ATCC 6538⁣^∗^ on smooth surfaces. AAHPA showed the highest log reduction of 4.72 against *E. coli*, whereas 0.2% H_2_O_2_ showed the highest log reduction of 6.95 against *S. aureus* (Figure 2). Log reduction of *E. coli* and *S. aureus* by AAHPA was statistically significant compared to 70% IPA, 1.5% CHG, and 10% NaOCl at *p* < 0.05 level.

### 3.4. Log and Percentage Reduction of Microbes on Rough Surfaces

**Figure 3:**
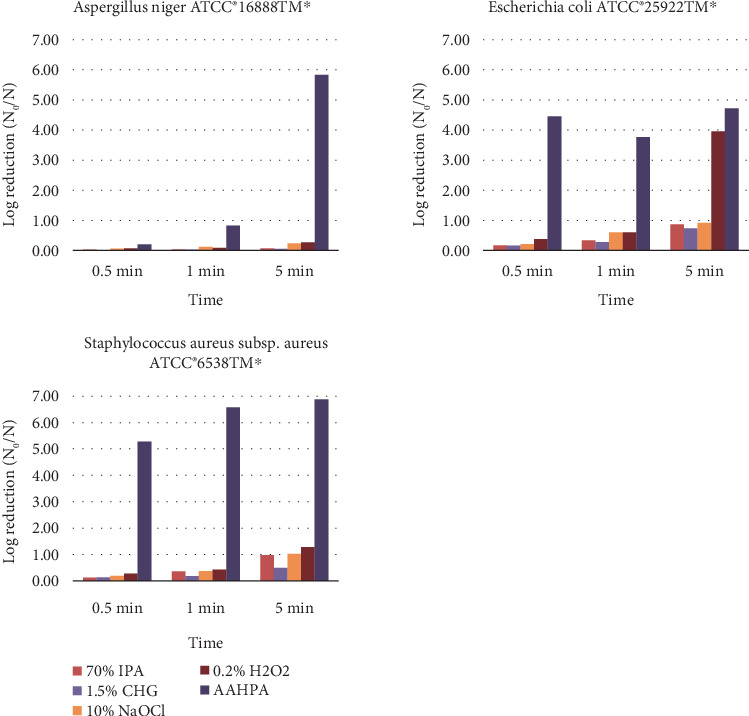
The log reduction of tested microbes and spores by five disinfectants on rough surfaces at different time intervals.

Table 3 shows the log and percentage reduction of tested microbes and spores by five disinfectants on rough surfaces. The disinfectant formulation AAHPA demonstrated a markedly higher percentage reduction at the *p* < 0.01 level compared to 70% IPA and 1.5% CHG and at *p* < 0.05 compared to 10% NaOCl and 0.2% H_2_O_2_ on rough surfaces against *A. niger* ATCC 16888⁣^∗^. At 5 min, AAHPA exhibited the greatest log and percentage reduction (5.83% and > 99.999%) on rough surfaces compared to all other tested disinfectants against *A. niger* (Figure 3). Against *E. coli* ATCC 25922⁣^∗^ and *S. aureus* subsp. *aureus* ATCC 6538⁣^∗^, the AAHPA showed the highest efficacy (Figure 3) on rough surfaces, and the log reduction of AAHPA was statistically significant at *p* < 0.01 level.

### 3.5. Effectiveness Against Microbes Based on Time and Surfaces

**Figure 4:**
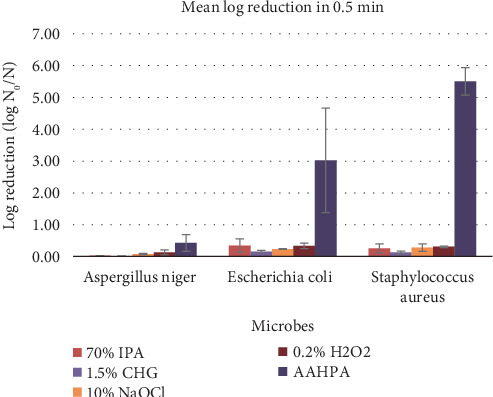
Effectiveness of disinfectants in 0.5 min.

**Figure 5:**
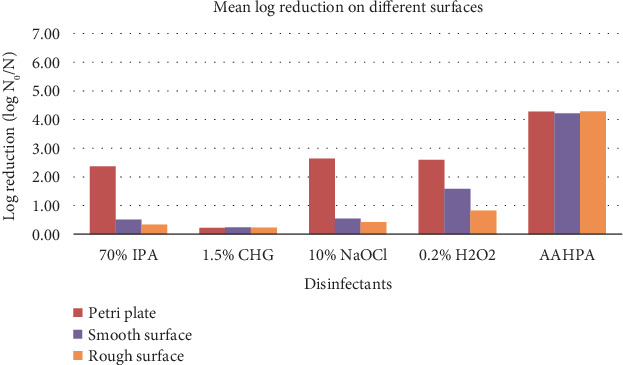
Surface efficacy of disinfectants (combined the log reductions of three contact times and the three organisms to measure the mean log reduction of each disinfectant on different surfaces).

The disinfectant formulation AAHPA showed remarkably high effectiveness against all three tested microbes and spores at 0.5 min compared to any other disinfectant on all surfaces (Figure 4). At the first 0.5 min, the *p* value of AAHPA was found to be less than 0.05 for *A. niger* and less than 0.01 for *E. coli* and *S. aureus* indicating the faster microbial decline efficacy of AAHPA compared to other disinfectants. The mean log reduction of tested microbes and spores at 0.5 min on all surfaces is tabulated in Table 4. For AAHPA, 0.2% H_2_O_2_ and 1.5% CHG, there were no significant mean differences between the log reductions of the three surfaces, indicating that these disinfectants are equally effective on all three surfaces. Moreover, compared to other disinfectants, AAHPA showed consistently higher log reduction on all surfaces against all tested microbes and spores (Tables 1, 2, and 3). Ten percent NaOCl and 70% IPA were more effective in petri plates (*p* < 0.05) than the other two surfaces (Figure 5).

## 4. Discussion

Investigating microbial susceptibility to various disinfectants across different surfaces and time intervals is crucial for advancing effective disinfection strategies. In this study, the assessment of five disinfectants against three distinct microbes—*A. niger* (spore), *E. coli* (gram-negative), and *S. aureus* (gram-positive)—provides valuable insights into the relative efficacy of these agents. The strains were selected to evaluate the efficacy of disinfectants against fungal spores as well as both gram-positive and gram-negative bacteria, covering diverse microbial threats.

Spores are the most stable form of bacteria and fungi that are highly resistant to heat and chemicals [33, 34]. According to this study AAHPA, the disinfectant formulation comprising 2.65% AA, 3% H_2_O_2_, and 0.1% PAA could kill the spores of *A. niger*, and so it may kill the microbes more effectively. The results of this study also prove this assumption. The results highlight that AAHPA consistently outperformed conventional disinfectants (70% IPA, 1.5% CHG, 10% NaOCl, and 0.2% H_2_O_2_) against tested fungal spores and bacteria. These findings align with prior studies that emphasized the fungicidal, sporicidal, and bactericidal properties of PAA and PAA-based formulations [35–37]. According to previous studies, chemical agents based on PAA inactivate yeasts, bacteria, fungi, and bacterial spores when exposed for 15 s–30 min at concentrations ranging from 0.02% to 1% [38–41]. The spores of *Bacillus megaterium*, *Bacillus amyloliquefaciens*, *Clostridioides difficile*, *Clostridium sporogenes*, and *Geobacillus thermophilus* were successfully prevented by these compounds [42–44] and removed biofilms formed by *Listeria monocytogenes* [45–47]. According to a study, while PAA is the primary agent responsible for spore inactivation, the presence of H_2_O_2_ enhances its efficacy. H_2_O_2_ compromises the integrity of the spore coat, which is a major barrier to disinfectants, thereby facilitating better penetration of PAA into the spore core. This increased penetration leads to more effective sporicidal activity [8].

In a study, a 4% equilibrium solution of H_2_O_2_ (27% *w*/*w*), AA (7.5% *w*/*w*), and PAA (5.0% *w*/*w*) was highly effective against planktonic *S. aureus*, with a 7 log reduction in 5 min, which is relatable to our findings [48]. Our current study found a 6.88 log reduction of *S. aureus* in 5 min by AAHPA on smooth surfaces, which supports the previous reports [48]. Moreover, at 5 min of exposure, 70% IPA, 10% NaOCl, and 0.2% H_2_O_2_ exhibited great antibacterial efficacy in several phases of the study, and these results are supported by previous studies [49–53]. The mixture of AAHPA stays in an equilibrium state, and so the concentrations of AAHPA remain constant at a specific environmental condition [10]. The presence of these three compounds together in a constant concentration in AAHPA may be the reason for its high effectiveness as a disinfectant.

CHG has been known as a versatile disinfectant, widely recognized for its broad-spectrum antimicrobial properties [54, 55]. Several studies reported that CHG is highly effective with > 4 log reduction against different microorganisms at low concentrations [52, 56–58]. Interestingly, in this study, the CHG showed the lowest microbial decline consistently on all surfaces among all the disinfectants. This may be due to the resistance of microbes against CHG. In some recent studies, several bacteria were found to be acquiring CHG resistance with significantly high minimal inhibitory concentrations [59, 60]. If so, more investigation should be carried out as CHG is still used for preventing healthcare-associated infections, and this can be life-threatening.

The time-dependent efficacy of AAHPA is noteworthy for quick microbial inactivation in practical disinfection scenarios. AAHPA's consistent effectiveness across petri plates, smooth surfaces, and rough surfaces shows the adaptability of this formulation to diverse environmental conditions. All microorganisms exhibited comparatively lower sensitivity to all the disinfectants on rough surfaces may be due to the presence of organic matter [48]. These results suggest that AAHPA can be used in hospitals and other healthcare-related places against infectious fungi and bacteria for rapid and effective disinfection purposes, such as surface and surgical tool disinfection. The study's unique focus on AAHPA's effectiveness against *A. niger* spores provides a valuable contribution beyond traditional bactericidal properties. While the study offers valuable insights, the limited strains of tested microbes might restrict the generalizability of results to a broader range of microbial threats. Additionally, evaluating disinfectants at a single concentration limits our understanding of potential concentration-dependent efficacy. Further research, building upon the findings of this study, can refine disinfection protocols, considering the microbial spectrum, surfaces encountered, and the specific fungicidal requirements.

## 5. Conclusion

The findings of this study signify the impact and versatility of the formulation AAHPA (a blend of AAHPA) as a potent disinfectant. Across various surfaces, AAHPA has demonstrated efficacy in rapidly killing a broad spectrum of microorganisms, including fungal spores and gram-positive and gram-negative bacteria, and thus positions AAHPA as a comprehensive solution for microbial control. This is particularly noteworthy in healthcare, where its application as a disinfectant could contribute to a substantial reduction in healthcare-associated infections. The adaptability and persistence of AAHPA on different surfaces and its rapid microbial inactivation make it a promising candidate for diverse scenarios, including emergency response situations where time efficiency is critical. Future research should focus on validating AAHPA in real-world scenarios, assessing long-term persistence, optimizing formulation, conducting clinical trials, and exploring its environmental impact.

## Figures and Tables

**Table 1:** Outcomes of surface disinfection activity on petri plate contaminated with different microbes using various disinfectants.

**Disinfectants name**		***Aspergillus niger* ATCC 16888**⁣^∗^			***Escherichia coli* ATCC 25922**⁣^∗^			***Staphylococcus aureus*** **subsp.** ***aureus* ATCC 6538**⁣^∗^		
		**Contact time (in minutes)**			**Contact time (in minutes)**			**Contact time (in minutes)**		
		**0.5 min**	**1** min	**5 min**	**0.5 min**	**1 min**	**5 min**	**0.5 min**	**1 min**	**5 min**
70% isopropyl alcohol (IPA)	i	3.7 × 10^3^	2 × 10^4^	1.8 × 10^5^	3.8 × 10^3^	5.4 × 10^3^	7.5 × 10^4^	5.4 × 10^5^	3.2 × 10^5^	1 × 10^6^
	ii	3.5 × 10^3^	1.8 × 10^4^	1.5 × 10^5^	1 × 10^3^	< 3	< 3	2.1 × 10^5^	< 3	< 3
	iii	0.024	0.046	0.079	0.58	3.73	4.88	0.41	5.51	6
	iv	5.41%	10.00%	16.67%	73.68%	99.98%	> 99.99%	61.11%	> 99.999%	99.9999%

1.5% chlorhexidine gluconate (CHG)	i	5.9 × 10^3^	3.8 × 10^4^	4.9 × 10^5^	1.8 × 10^3^	2.8 × 10^4^	7.4 × 10^3^	4.7 × 10^4^	1.9 × 10^5^	4.9 × 10^5^
	ii	5.7 × 10^3^	3.5 × 10^4^	4.3 × 10^5^	1.4 × 10^3^	1.3 × 10^4^	1 × 10^3^	3.9 × 10^4^	1.2 × 10^5^	2.3 × 10^5^
	iii	0.015	0.036	0.057	0.11	0.33	0.87	0.081	0.20	0.33
	iv	3.39%	7.89%	12.25%	22.22%	53.57%	86.49%	17.02%	36.84%	53.06%

10% sodium hypochlorite (NaOCl)	i	2.5 × 10^5^	3.2 × 10^5^	7.9 × 10^5^	4.9 × 10^3^	6.3 × 10^3^	7.6 × 10^4^	5.2 × 10^5^	5.2 × 10^6^	9.2 × 10^6^
	ii	2 × 10^5^	1.9 × 10^5^	3.1 × 10^5^	2.8 × 10^3^	< 3	< 3	2 × 10^5^	< 3	< 3
	iii	0.097	0.23	0.41	0.24	3.80	4.88	0.42	6.72	6.96
	iv	20%	40.63%	60.76%	42.86%	99.98%	> 99.99%	61.54%	> 99.9999%	> 99.9999%

0.2% hydrogen peroxide (H_2_O_2_)	i	2.8 × 10^4^	5.9 × 10^5^	7.9 × 10^5^	3.8 × 10^4^	2.1 × 10^4^	6.5 × 10^4^	2.1 × 10^5^	5.9 × 10^5^	8.9 × 10^6^
	ii	1.7 × 10^4^	3.1 × 10^5^	2.9 × 10^5^	1 × 10^4^	< 3	< 3	1 × 10^5^	< 3	< 3
	iii	0.22	0.28	0.44	0.23	4.32	4.81	0.32	5.77	6.95
	iv	39.30%	47.50%	63.30%	41.54%	> 99.99%	> 99.99%	52.38%	> 99.999%	> 99.9999%

A mixture of acetic acid, hydrogen peroxide, and peracetic acid (AAHPA)	i	6.3 × 10^4^	3.8 × 10^4^	6.9 × 10^5^	4.7 × 10^4^	1.9 × 10^3^	3.2 × 10^3^	1 × 10^6^	3.8 × 10^6^	5.9 × 10^6^
	ii	1.2 × 10^4^	< 3	< 3	2.8 × 10^3^	< 3	< 3	< 3	< 3	< 3
	iii	0.72	4.58	5.84	1.23	3.28	3.51	6	6.58	6.77
	iv	80.95%	> 99.99%	> 99.999%	94.04%	99.95%	99.97%	99.9999%	> 99.9999%	> 99.9999%

*Note:* i: microbial count (CFU/mL) on the surface prior to disinfection. ii: microbial count (CFU/mL) on the surface after disinfection. iii: log decline in CFU upon disinfection. iv: percentage of killing following disinfection.

**Table 2:** Outcomes of surface disinfection activity on smooth surfaces contaminated with different microbes using various disinfectants.

**Disinfectants name**		***Aspergillus niger* ATCC 16888**⁣^∗^			***Escherichia coli* ATCC 25922**⁣^∗^			***Staphylococcus aureus* subsp. *aureus* ATCC 6538**⁣^∗^		
		**Contact time (in minutes)**			**Contact time (in minutes)**			**Contact time (in minutes)**		
		**0.5** min	**1** min	**5 min**	**0.5 min**	**1 min**	**5 min**	**0.5 min**	**1 min**	**5 min**
70% isopropyl alcohol (IPA)	i	3.7 × 10^4^	5.4 × 10^4^	2.5 × 10^5^	3.2 × 10^4^	9.4 × 10^4^	8.2 × 10^4^	7.1 × 10^5^	7.4 × 10^5^	8.5 × 10^5^
	ii	3.5 × 10^4^	4.9 × 10^4^	2.1 × 10^5^	1.7 × 10^4^	3.4 × 10^4^	2.7 × 10^3^	4.3 × 10^5^	2.9 × 10^5^	2.1 × 10^4^
	iii	0.024	0.042	0.076	0.27	0.44	1.48	0.22	0.41	1.61
	iv	5.41%	9.26%	16.00%	46.88%	63.83%	96.71%	39.44%	60.81%	97.53%

1.5% chlorhexidine gluconate (CHG)	i	4.9 × 10^3^	5.7 × 10^3^	7.9 × 10^5^	3.2 × 10^3^	5.9 × 10^3^	7.9 × 10^3^	3.9 × 10^5^	5.9 × 10^6^	7.5 × 10^6^
	ii	4.7 × 10^3^	5.2 × 10^3^	7.8 × 10^5^	2.1 × 10^3^	3.1 × 10^3^	1.8 × 10^3^	2.7 × 10^5^	3.4 × 10^6^	2.1 × 10^6^
	iii	0.018	0.040	0.065	0.18	0.28	0.64	0.16	0.24	0.55
	iv	4.08%	8.77%	13.92%	34.38%	47.46%	77.21%	30.77%	42.37%	72%

10% sodium hypochlorite (NaOCl)	i	7.9 × 10^4^	3.2 × 10^5^	4.9 × 10^4^	5.9 × 10^3^	7.8 × 10^4^	5.7 × 10^4^	4.7 × 10^5^	3.9 × 10^5^	3.8 × 10^6^
	ii	6.5 × 10^4^	2.1 × 10^5^	2.5 × 10^4^	3.4 × 10^3^	1.9 × 10^4^	1.6 × 10^3^	2.8 × 10^5^	1.5 × 10^5^	2.1 × 10^6^
	iii	0.085	0.18	0.29	0.24	0.61	1.55	0.22	0.42	1.26
	iv	17.72%	34.38%	48.98%	42.37%	75.64%	97.19%	40.42%	61.54%	94.47%

0.2% hydrogen per oxide (H_2_O_2_)	i	7.3 × 10^3^	6.5 × 10^3^	5.1 × 10^3^	3.2 × 10^3^	9.8 × 10^3^	3.2 × 10^4^	5.4 × 10^6^	3.9 × 10^6^	8.9 × 10^6^
	ii	5.8 × 10^3^	3.9 × 10^3^	2.4 × 10^3^	1.3 × 10^3^	1.5 × 10^3^	< 3	2.6 × 10^6^	1 × 10^6^	< 3
	iii	0.10	0.22	0.33	0.39	0.82	4.51	0.32	0.59	6.95
	iv	20.55%	40%	52.94%	59.38%	84.69%	> 99.99%	51.85%	74.36%	> 99.9999%

A mixture of acetic acid, hydrogen peroxide, and peracetic acid (AAHPA)	i	2.9 × 10^4^	7.5 × 10^4^	9.7 × 10^5^	2.5 × 10^3^	4.9 × 10^4^	3.2 × 10^4^	1.7 × 10^5^	5.3 × 10^5^	9.1 × 10^5^
	ii	1.3 × 10^4^	8.3 × 10^3^	< 3	< 3	< 3	< 3	< 3	< 3	7.5 × 10^6^
	iii	0.35	0.96	5.99	3.39	4.69	4.72	5.23	5.72	6.88
	iv	55.17%	88.93%	> 99.999%	99.96%	> 99.99%	> 99.99%	> 99.999%	> 99.999%	> 99.9999%

*Note:* i: microbial count (CFU/mL) on the surface prior to disinfection. ii: microbial count (CFU/mL) on the surface after disinfection. iii: log decline in CFU upon disinfection. iv: percentage of killing following disinfection.

**Table 3:** Outcomes of surface disinfection activity on rough surfaces contaminated with different microbes using various disinfectants.

**Disinfectants name**		***Aspergillus niger* ATCC 16888**⁣^∗^			***Escherichia coli* ATCC 25922**⁣^∗^			***Staphylococcus aureus* subsp. *aureus* ATCC 6538**⁣^∗^		
		**Contact time (in minutes)**			**Contact time (in minutes)**			**Contact time (in minutes)**		
		**0.5** min	**1 min**	**5 min**	**0.5 min**	**1 min**	**5 min**	**0.5 min**	**1 min**	**5 min**
70% isopropyl alcohol (IPA)	i	1.5 × 10^4^	7.9 × 10^3^	3.5 × 10^5^	9.9 × 10^3^	7.6 × 10^4^	8.9 × 10^4^	3.2 × 10^6^	4.9 × 10^6^	7.9 × 10^6^
	ii	1.4 × 10^4^	7.2 × 10^3^	3.0 × 10^5^	6.7 × 10^3^	3.5 × 10^4^	1.2 × 10^4^	2.4 × 10^6^	2.1 × 10^6^	8.2 × 10^5^
	iii	0.030	0.040	0.067	0.17	0.34	0.87	0.12	0.37	0.98
	iv	6.67%	8.86%	14.29%	32.32%	53.95%	86.52%	25%	57.14%	89.62%

1.5% chlorhexidine gluconate (CHG)	i	3.2 × 10^4^	4.9 × 10^5^	8.4 × 10^5^	3.5 × 10^4^	6.5 × 10^4^	9.8 × 10^3^	5.8 × 10^5^	6.8 × 10^5^	1.2 × 10^6^
	ii	3.1 × 10^4^	4.6 × 10^5^	7.5 × 10^5^	2.4 × 10^4^	3.4 × 10^4^	1.8 × 10^3^	4.2 × 10^5^	4.5 × 10^5^	3.8 × 10^5^
	iii	0.014	0.027	0.049	0.16	0.28	0.74	0.14	0.18	0.50
	iv	3.13%	6.12%	10.71%	31.43%	47.69%	81.63%	27.59%	33.82%	68.33%

10% sodium hypochlorite (NaOCl)	i	7.3 × 10^4^	2.5 × 10^5^	5.3 × 10^5^	4.8 × 10^4^	3.9 × 10^4^	5.3 × 10^4^	6.4 × 10^5^	7.3 × 10^5^	5.3 × 10^6^
	ii	6.4 × 10^4^	1.9 × 10^5^	3.1 × 10^5^	2.9 × 10^4^	9.8 × 10^3^	6.4 × 10^3^	4.1 × 10^5^	3.1 × 10^5^	4.9 × 10^5^
	iii	0.057	0.12	0.23	0.22	0.60	0.92	0.19	0.37	1.03
	iv	12.33%	24.00%	41.51%	39.58%	74.87%	87.93%	35.94%	57.53%	90.76%

0.2% hydrogen peroxide (H_2_O_2_)	i	7.4 × 10^3^	8.9 × 10^4^	9.2 × 10^4^	5.3 × 10^3^	8.5 × 10^3^	9.2 × 10^3^	8.9 × 10^6^	7.8 × 10^6^	5.9 × 10^6^
	ii	6.3 × 10^3^	7.2 × 10^4^	5 × 10^4^	2.2 × 10^3^	2.1 × 10^3^	< 3	4.7 × 10^6^	2.9 × 10^6^	3.1 × 10^5^
	iii	0.070	0.092	0.26	0.38	0.61	3.96	0.28	0.43	1.28
	iv	14.87%	19.10%	45.65%	58.49%	75.29%	99.99%	47.19%	62.82%	94.75%

A mixture of acetic acid, hydrogen peroxide, and peracetic acid (AAHPA)	i	8.6 × 10^5^	3.9 × 10^4^	6.7 × 10^5^	2.8 × 10^4^	5.9 × 10^3^	5.3 × 10^4^	1.9 × 10^5^	3.8 × 10^6^	7.5 × 10^6^
	ii	5.3 × 10^5^	5.8 × 10^3^	< 3	< 3	< 3	< 3	< 3	< 3	< 3
	iii	0.21	0.83	5.83	4.45	3.77	4.72	5.28	6.58	6.88
	iv	38.37%	85.13%	> 99.999%	> 99.99%	99.98%	> 99.99%	> 99.999%	> 99.9999%	> 99.9999%

*Note:* i: microbial count (CFU/mL) on the surface prior to disinfection. ii: microbial count (CFU/mL) on the surface after disinfection. iii: log decline in CFU upon disinfection. iv: percentage of killing following disinfection.

**Table 4:** Mean log reduction of tested microbes at 0.5 min on all surfaces (Figure 4).

**Microorganisms**	**M** **e** **a** **n** log **reduction** ± **standard** **deviation**				
	**70% IPA**	**1.5% CHG**	**10% NaOCl**	**0.2% H**_**2**_ **O**_**2**_	**AAHPA**
*Aspergillus niger*	0.03 ± 0.003	0.02 ± 0.002	0.08 ± 0.02	0.13 ± 0.08	0.43 ± 0.26
*Escherichia coli*	0.34 ± 0.21	0.15 ± 0.04	0.23 ± 0.01	0.34 ± 0.09	3.02 ± 1.64
*Staphylococcus aureus*	0.25 ± 0.15	0.13 ± 0.04	0.28 ± 0.12	0.30 ± 0.02	5.50 ± 0.43

## Data Availability

The data that support the findings of this study are available from the corresponding author upon reasonable request.

## Supporting Information

Supporting Information 1 Contains outcomes of all tables and the hypothetical conclusion of this study.

Supporting Information 2 Contains figures.
